# Preliminary Attempt to Predict Risk of Invasive Pulmonary Aspergillosis in Patients with Influenza: Decision Trees May Help?

**DOI:** 10.3390/antibiotics9100644

**Published:** 2020-09-26

**Authors:** Valeria Bellelli, Guido Siccardi, Livia Conte, Luigi Celani, Elena Congeduti, Cristian Borrazzo, Letizia Santinelli, Giuseppe Pietro Innocenti, Claudia Pinacchio, Giancarlo Ceccarelli, Mario Venditti, Gabriella d’Ettorre

**Affiliations:** 1 Department of Public Health and Infectious Diseases, University of Rome Sapienza, 00182 Rome, Italy; valeria.bellelli@uniroma1.it (V.B.); guido.siccardi@uniroma1.it (G.S.); liv.conte@gmail.com (L.C.); luigi.celani@uniroma1.it (L.C.); cristian.borrazzo@uniroma1.it (C.B.); letizia.santinelli@uniroma1.it (L.S.); giuseppepietro.innocenti@uniroma1.it (G.P.I.); claudia.pinacchio@uniroma1.it (C.P.); giancarlo.ceccarelli@uniroma1.it (G.C.); mario.venditti@uniroma1.it (M.V.); 2 Electrical Engineering Mathematics and Computer Science, Delft University of Technology, 2628 XE Delft, The Netherlands; e.congeduti@tudelft.nl

**Keywords:** invasive pulmonary aspergillosis, influenza, machine learning, decision trees, Italy, EORTC/MSG, antifungal drugs, risk score

## Abstract

Invasive pulmonary aspergillosis (IPA) is typically considered a disease of immunocompromised patients, but, recently, many cases have been reported in patients without typical risk factors. The aim of our study is to develop a risk predictive model for IPA through machine learning techniques (decision trees) in patients with influenza. We conducted a retrospective observational study analyzing data regarding patients diagnosed with influenza hospitalized at the University Hospital “Umberto I” of Rome during the 2018-2019 season. We collected five IPA cases out of 77 influenza patients. Although the small sample size is a limit, the most vulnerable patients among the influenza-infected population seem to be those with evidence of lymphocytopenia and those that received corticosteroid therapy.

## 1. Introduction

The association between invasive pulmonary aspergillosis (IPA) and influenza has begun to attract particular attention over the last 20 years. It represents a worldwide phenomenon, reported in at least 16 countries from across Europe, Asia, and the United States of America [1]. Since IPA often has severe consequences, with a mortality rate between 50 and 90%, especially if the diagnosis is delayed [2,3], it is essential to identify patients at higher risk so that they can be monitored closely, and prophylaxis can be considered. Therefore, we designed a retrospective observational study on hospitalized patients diagnosed with influenza during the 2018-2019 winter season with the aim of developing a risk predictive model for IPA through machine learning techniques (decision trees) [4,5].

## 2. Results

During the 2018-2019 flu season, 104 episodes of influenza among patients hospitalized at the “Umberto I” hospital were recorded. Twenty-seven patients presented the exclusion criteria; therefore, 77 patients were included in the study (Table 1) and five (6.5%) out of these developed IPA superinfections. In one case, an autopsy was performed with a histopathological diagnosis; in three cases, the mycological criterion was the positivity of GM on BAL (in all three cases the culture for *Aspergillus* was negative), in one case GM on serum. In all cases, a worsening of respiratory exchanges was observed, and two patients also presented a refractory fever. Thirty-four episodes were classified as mild influenza (44%) and 43 as severe flu (56%). Univariate analysis of preexisting and hospitalization-related risk factors for IPA are presented in Table 2 and Table 3. All cases of IPA were patients with severe flu (Table 4).

We have subsequently developed a risk predictive model of IPA through a machine learning technique: decision-making trees, with the aim of providing clinicians with a tool capable of accurately identifying IPA high-risk patients, in order to monitor them closely and evaluate antifungal prophylaxis. 

**Figure 1:**
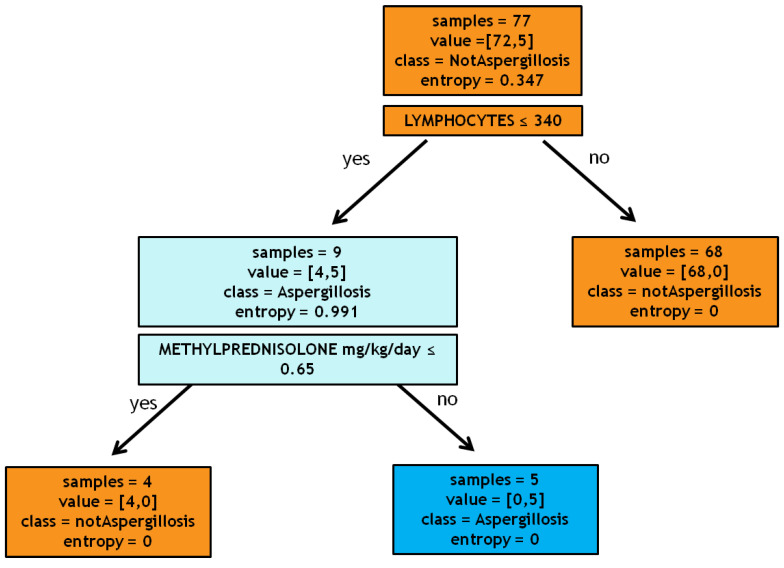
Decision tree.

The predictive model has highlighted two variables as decisive in risk assessment: lymphocytes count ≤340/µL and methylprednisolone administration >0.65 mg/kg/day, as shown in Figure 1 (see Section 4.3 Statistical analysis for more details). A 10-fold cross-validation [4] test revealed 87.5% as the average sensitivity and 96.4% as the average specificity.

## 3. Discussion

The innovative aspect of our study is the development of a methodology based on a machine learning technique, the decision tree, for building a risk predictive model for IPA in patients with influenza. Since IPA is a disease with many possible complications and often poor prognosis, a risk predictive score would be a tool of crucial importance. This predictive model, although built on a very small dataset, highlighted two variables as determinants in the assessment of risk: lymphocytopenia at diagnosis of influenza with a lymphocyte count ≤340/µL and administration of methylprednisolone during hospitalization at a dosage of more than 0.65 mg/kg/day. These results, although preliminary, seem to be of particular interest. Various studies have shown the role of lymphocytes in invasive *Aspergillus* infections, especially the Th1 and Th17 subpopulations [6,7], and furthermore, lymphocytopenia was recognized among the independent risk factors for pulmonary superinfections [8]. Another study proposes virus-induced lymphocytopenia among the risk factors associated with IPA in patients with influenza [9]. The degree of lymphocytopenia is an expression of an increased apoptotic rate in the context of an abnormal anti-inflammatory response with immunoparalysis and anergy of the immune system, a condition that typically occurs in severe influenza infection, as well as increased recruitment at extensively inflamed lung tissue. The role of corticosteroids as a risk factor for invasive fungal infections has been known for many years [10]. They alter the immune system by affecting different pathways [11]. In addition to immune dysfunction, which certainly plays a key role in the pathogenesis of invasive aspergillosis, it has been hypothesized that glucocorticoids have a direct effect on *Aspergillus* species, inducing their growth [12]. Our study has a number of limitations: (1) the small sample number does not allow us to build a score but only to identify the factors related to an increased risk of superinfection; (2) the retrospective nature makes the study less precise and more susceptible to error; (3) in this study, the decision tree is highly based on the “use of corticosteroids”. However, several previous studies reported that a number of the patients developing IPA did not use corticosteroids: in this case, the decision tree could have significant limitations. One of the innovative aspects of our study is to propose a methodology for the prediction of the risk through machine learning techniques, whose applications remain almost unexplored in this field.

## 4. Materials and Methods 

### 4.1. Study Design, Inclusion and Exclusion Criteria, and Definitions

We conducted a retrospective observational study analyzing data regarding patients diagnosed with influenza hospitalized at the University Hospital “Umberto I” of Rome during the 2018-2019 season. The first case dates back to the 3rd of January and the last to the 23rd of April 2019. Exclusion criteria were history of chronic or invasive aspergillosis or immunosuppression according to EORTC/MSG criteria [13]: a recent history of neutropenia (<500 neutrophils/mm3) for >10 days, receipt of an allogeneic stem cell transplant, prolonged use of corticosteroids at a mean minimum dose of 0.3 mg/kg/die of prednisone equivalent for >3 weeks, treatment with other recognized T-cell immunosuppressants, and inherited severe immunodeficiency. Influenza virus infection was diagnosed if viral RNA on respiratory samples resulted as positive. A real-time PCR technique (CEPHEID kit) was used with primers for the RNA of influenza A, B, and H1N1 subtypes. This analysis was requested by the ward physicians in patients with suspected influenza infection. To define a case of IPA, the criteria of the modified AspICU algorithm were adopted [14]. One or more of the following mycological criteria had to be present: histopathology or direct microscopic evidence of dichotomous septate hyphae with a positive culture for *Aspergillus* from tissue; a positive *Aspergillus* culture from a bronchoalveolar lavage (BAL); a galactomannan optical index on BAL of ≥1; a galactomannan optical index on serum of ≥0.5; associated with at least one of the following clinical criteria: fever refractory to at least 3 days of appropriate antibiotic therapy; recrudescent fever after a period of defervescence of at least 48 h while still on antibiotics and without other apparent cause; dyspnea; hemoptysis; pleural friction, rub or chest pain; worsening respiratory insufficiency in spite of appropriate antibiotic therapy and ventilatory support; associated with the presence of the radiographic criteria: any pulmonary infiltrate detected at RX or CT.

### 4.2. Population Analysis

Demographic, clinical, and anamnestic patients’ data were collected from the patient’s medical records, from the access to first aid until discharge or death, all discharged patients were contacted three months after the dismission in order to confirm the absence of new hospital admission. In particular, age, sex, weight, ethnicity, smoking, alcoholic habits, comorbidities, and the Charlson Comorbidity Index were recorded. For each patient, at the diagnosis of influenza, type of influenza virus, community or nosocomial-acquisition, the value of white blood cells, neutrophils, lymphocytes and CRP (C reactive protein), SOFA score (sequential organ failure assessment), radiographic findings, need for oxygen supplementation (via Venturi mask, non-invasive mechanical ventilation or invasive mechanical ventilation), were recorded as well as the eventual need for ECMO support (extracorporeal membrane oxygenation) and hemodialysis during hospitalization. We divided patients diagnosed with influenza into two groups: patients with influenza who developed IPA, and patients who did not undergo this superinfection, we therefore developed a risk predictive model for IPA through machine learning techniques (decision trees), to try to identify IPA high-risk patients. We also assessed the risk factors and the clinical impact associated with IPA. In a first analysis, we compared patients with IPA with patients without. We defined “severe flu” as all episodes of influenza that required oxygen therapy for at least 48 h. The study protocol was approved by the Hospital Ethics Commission and, considering the retrospective nature of the study, the request for informed consent to patients was omitted.

### 4.3. Statistical Analysis

We developed a decision tree-based predicting model [4,5]. The algorithm has been coded in Python 3.5. As splitting criteria, we used entropy. In order to assess the quality of the model, we performed a 10-fold cross-validation test, considering the sensitivity and specificity as performance measures.

Precisely, the algorithm processes the database containing all patients with influenza through a progressive sequence of tests. These tests, with a positive or negative outcome for the categorical variable and greater or smaller than a given threshold for the continuous variables, were performed by the algorithm on all the instances in the database. Subsequently, we asked the algorithm to provide the variables that split the samples into classes (IPA and non-IPA). For our specific cohort, as the first test, the algorithm selected the variable lymphocytes ≤340/µL; this variable divides the root (i.e., the set of all patients) into two leaves: a pure leaf, patients with lymphocytes more than 340/µL, consisting of 68 all non-IPA patients, and an impure leaf, patients with lymphocytes less than 340/µL, consisting of 4 non-IPA patients and 5 IPA patients. Then, we asked the algorithm to choose a new test, namely the variable that most divided IPA from non-IPA patients among this population of 9 subjects. The algorithm identified the administration of methylprednisolone at a dosage greater than 0.65 mg/kg/day. Eventually, we obtained two pure leaves: one containing the 4 patients taking less than 0.65 mg/kg/day, none of which were affected by IPA, and one containing 5 patients taking more than 0.65 mg/kg/day, all with the diagnosis of IPA. The new cases of influenza will be processed with the built model to predict the probability of developing aspergillosis as follows: the patient will be tested according to the sequence of questions identified by the tree and eventually lie in a leaf that will determine the class (IPA or non-IPA). Other statistical analyses were performed using Microsoft Excel (Office 2019) or Statistical Program for the Social Sciences (SPSS) version 20. To assess the normal distribution, the coefficients of kurtosis, skewness, and histogram plots were used. The data, unless otherwise stated, were reported as median with interquartile range (IQR: 25th and 75th percentile) for continuous variables and as simple frequencies (*n*) and proportions (or percentages) for dichotomous variables. Group comparisons in univariate analysis were made using the Mann–Whitney or Kruskal–Wallis tests for continuous variables. The Fisher’s exact or Chi-square tests were used as appropriate to test group differences of proportions. Lastly, parameters that were statistically significant in a univariate way were used in the logistic regression analysis to estimate adjusted odds ratios (ORs) and 95% confidence intervals (95% CI) for the risk factors associated with the development of IPA, including the confounding factors (age, gender, etc.). A *p*-value of less than 0.05 was considered statistically significant.

## 5. Conclusions

From our data, we confirm the risk of developing IPA during influenza. The onset of this complication severely conditions the prognosis and results in high mortality rates. The most vulnerable patients among the influenza-infected population are those that present the following risk factors: smoking habit, diagnosis of COPD, evidence of lymphocytopenia, and administration of corticosteroids. A methodology for the prediction of IPA risk that uses a technique of machine learning, decision trees, seems to provide interesting results but would need to be corroborated and possibly validated in larger patient cohorts.

## Figures and Tables

**Table 1:** Population characteristics.

**Population Characteristics: Continuous Variables**			
**Total (77 pt)**	**Median**	**IQR 25%**	**IQR 75%**
Age (years)	56.5	26.8	74.5
Weight (kg)	68	55	75
† WBC (cell/µL)	8640	5250	10,770
Neutrophils (cell/µL)	5730	2990	9190
Neutrophils %	78	6	88
Lymphocytes (cell/µL)	810	560	1400
Lymphocytes %	11	6	29
‡ CRP µg/L	39,150	10,250	67,850
CHARLSON	2	0	5
SOFA score	1	0	3
Methylprednisolone mg/kg/day	0.6	0.5	1
§ LOS (days)	12	7	34
**Population Characteristics: Categorical Variables**			
	**Patients**	**Patients (%)**	
Male	39	50.6	
Female	38	49.4	
Caucasian	70	90.9	
Asian	5	6.5	
African	2	2.6	
Smoker	35	45.5	
Alcohol	4	5.2	
No comorbidities	17	22.1	
† Ischemic/CHF	21	27.3	
‡ COPD	14	18.2	
§ CKD	6	7.8	
Cirrhosis	2	2.6	
Diabetes type 2	15	19.5	
Neurologic diseases	20	26	
Malignancies	6	7.8	
Autoimmune disorders	5	6.5	
Psychiatric diseases	8	10.4	
Pregnancy	2	2.6	
Influenza A	48	62.3	
Influenza H1N1	29	37.7	
Community-acquired	67	87	
Hospital-acquired	10	13	
Nasal swab	73	94.8	
Pharyngeal swab	3	3.9	
Nasopharyngeal aspirate	1	1.3	
Pneumonia (chest X-ray)	46	59.7	
Pleural effusion	17	22.1	
No oxygen supplementation	34	44.1	
Oxygen supplementation	24	31.2	
¶ NIV	6	7.8	
£ IMV	13	16.9	
$ ECMO	2	2.6	
Hemodialysis	4	5.2	
% ICU/emergency	17	22.1	
& BSI	12	15.6	
Lung superinfection	7	9.1	
Fungal superinfection	6	7.8	
IPA	5	6.5	
Oseltamivir	66	85.7	
Antifungal	19	24.7	
Antibiotic	59	76.6	
Corticosteroid	34	44.1	
Death	11	14.3	

† WBC: White Blood Cells; ‡ CRP: C Reactive Protein; § LOS: Length of Stay; † CHF: Chronic Cardiac Failure; ‡ COPD: Chronic Obstructive Pulmonary Disease; § CKD: Chronic Kidney Disease; ¶ NIV: Non-Invasive Ventilation; £ IMV: Invasive Mechanical Ventilation; $ ECMO: Extra Corporeal membrane Oxygenation; % ICU: Intensive Care Unit; & BSI: Blood Stream Infection; ^ IPA: Invasive Pulmonary Aspergillosis.

**Table 2:** Preexisting risk factors associated with IPA in influenza patients.

Risk Factors	Influenza and IPA	Influenza	*p-*Value
	(*n* = 5)	(*n* = 72)	
Age (years)	59 (58–71)	53 (26.5–75)	0.723
Male	3 (60)	36 (50)	0.667
Weight (kg)	80 (70–80)	65.7 (54.3–75)	0.740
Caucasian	4 (80)	66 (92)	0.381
Smoker	5 (100)	30 (42)	**0.0124**
Alcohol abuse	0	4 (5)	0.603
Pregnancy	0	2 (3)	0.706
† Ischemic/CHF	2 (40)	19 (25)	0.451
‡ COPD	4 (80)	10 (13)	**<0.001**
CKD	1 (20)	5 (7)	0.267
Cirrhosis	0	2 (3)	0.717
Diabetes type 2	0	15 (21)	0.259
Neurologic diseases	1 (20)	19 (26)	0.911
Malignancies	0	6 (8)	0.505
Autoimmune disorders	1 (20)	4 (6)	0.210
Psychiatric diseases	1 (20)	7 (9)	0.431
Charlson comorbidity index	2 (1–6)	2 (0–5)	0.889

† CHF: Chronic Heart Failure; ‡ COPD: Chronic Obstructive Pulmonary Disease; § CKD: Chronic Kidney Disease. Significant *p* values are highlighted in bold.

**Table 3:** Hospitalization-related risk factors associated with IPA in influenza patients.

Risk Factors	Influenza and IPA	Influenza	*p-*Value
	(*n* = 5)	(*n* = 72)	
Influenza A	2 (40)	46 (64)	0.287
Influenza H1N1	3 (60)	26 (36)	0.287
Community acquired	4 (80)	63 (87)	0.443
Hospital acquired	1 (20)	9 (13)	0.532
† WBC (cell/mmc)	9820 (8640–10,000)	8315 (5147.5–10,785)	0.529
Neutrophils (cell/µL)	9181 (7640.0–9400)	5550 (2920–9175)	0.328
Lymphocytes (cell/µL)	300 (274–304)	875 (587.5–1467.5)	**<0.001**
‡ CRP µg/L	53,650 (49,325–61,000)	35,650 (10,150–69,550)	0.332
§ SOFA score	5 (3–13)	1 (0–2)	0.095
Pneumonia chest X ray	5 (100)	41 (53)	**0.042**
Severe flue	5 (100)	38 (49)	**0.029**
Corticosteroid	5 (100)	29 (40)	**0.009**
Methylprednisolone mg/kg/day	1 (1–1.1)	0.6 (0.5–0.7)	**<0.001**

† WBC: White Blood Cell; ‡ CRP: C Reactive Protein; § SOFA: Sequential Organ Failure Assessment. Significant *p* values are highlighted in bold.

**Table 4:** Clinical impact of *Aspergillus* superinfection in patients with influenza.

Clinical Variables	Influenza and IPA (*n* = 5)	Influenza (*n* = 72)	*p-*Value
† BSI	3 (60)	9 (13)	**0.006**
Bacterial lung superinfection	1 (20)	6 (83)	**<0.001**
Pneumocystosis	0	1 (1)	0.792
‡ IMV	5 (100)	8 (11)	**<0.001**
ECMO	1 (20)	1 (1)	**0.012**
Hemodialysis	2 (40)	2 (3)	**0.003**
LOS (days)	37 (33–49)	11 (7–30)	0.159
Death	5 (100)	6 (8)	**<0.001**

† BSI: Blood Stream Infection; ‡ IMV: Invasive Mechanical Ventilation; § ECMO: Extra Corporeal Membrane Oxygenation; ¶ LOS: Length of Stay. Significant *p* values are highlighted in bold.
